# The Importance of Hydration in Body Composition Assessment in Children Aged 6-16 Years

**DOI:** 10.1016/j.jocd.2020.12.004

**Published:** 2021

**Authors:** Laura P.E. Watson, Katherine S. Carr, Elise R. Orford, Michelle C. Venables

**Affiliations:** 1 National Institute Health Research Cambridge Clinical Research Facility, Addenbrooke's Hospital, Cambridge, United Kingdom; 2 Stable Isotope Laboratory, National Institute for Health Research Cambridge Biomedical Research Centre Nutritional Biomarker Laboratory, MRC Epidemiology Unit and Wellcome-MRC Institute of Metabolic Science, Metabolic Research Laboratories, University of Cambridge, Cambridge, United Kingdom

**Keywords:** Densitometry, cross-calibration equations, deuterium dilution, tissue hydration, paediatrics, ADP, air displacement plethysmography, BM, bone mass, BMC, whole body bone mineral content, BV, body volume, CRF, clinical research facility, DFFM, fat free mass density, DXA, dual energy X-ray absorptiometry, FFM, fat free mass, FM, fat mass, FRC, functional residual capacity, HFFM, fat free mass hydration, LOA, limits of agreement, LST, lean soft tissue, SA, air next to the skin, SAA, SA artefact, SDS, standard deviation score, TBW, total body water, TGC, thoracic gas volume, TV, tidal volume, V-SMOW, Vienna standard mean ocean water, 4-C, four-component

## Abstract

Body composition is associated with many noncommunicable diseases. The accuracy of many simple techniques used for the assessment of body composition is influenced by the fact that they do not take into account tissue hydration and this can be particularly problematic in paediatric populations. The aims of this study were: (1) to assess the agreement of two dual energy X-ray absorptiometry (DXA) systems for determining total and regional (arms, legs, trunk) fat, lean, and bone mass and (2) to compare lean soft tissue (LST) hydration correction methods in children. One hundred and twenty four healthy children aged between 6 and 16 years old underwent DXA scans using 2 GE healthcare Lunar systems (iDXA and Prodigy). Tissue hydration was either calculated by dividing total body water (TBW), by 4-component model derived fat free mass (HFFM_TBW_) or by using the age and sex specific coefficients of Lohman, 1986 (HFFM_Lohman_) and used to correct LST. Regression analysis was performed to develop cross-calibration equations between DXA systems and a paired samples *t*-test was conducted to assess the difference between LST hydration correction methods. iDXA resulted in significantly lower estimates of total and regional fat and lean mass, compared to Prodigy. HFFM_TBW_ showed a much larger age/sex related variability than HFFM_Lohman_. A 2.0 % difference in LST was observed in the boys (34.5 kg *vs* 33.8 kg respectively, p < 0.05) and a 2.5% difference in the girls (28.2 kg *vs* 27.5 kg respectively, p < 0.05) when corrected using either HFFM_TBW_ or HFFM_Lohman._ Care needs to be exercised when combining data from iDXA and Prodigy, as total and regional estimates of body composition can differ significantly. Furthermore, tissue hydration should be taken into account when assessing body composition as it can vary considerably within a healthy paediatric population even within specific age and/or sex groups.

## Introduction

The accurate assessment of body composition in children is an important facet in determining typical growth. As excess accumulation of fat mass (FM) is associated with insulin resistance, obesity, and cardiovascular risk in later childhood and adulthood *(**1**)* and fat free mass (FFM) is directly related to energy expenditure and glucose handling, a true estimation of these components can be important. The ability to measure accurately body composition becomes even more pertinent as in some clinical conditions associated with protein energy wasting e.g., end-stage renal disease and severe acute malnutrition, the loss of fat-free mass can be directly related to poor clinical outcomes and mortality *(**2**,* *3**).*

Dual energy X-ray absorptiometry (DXA) is now a widely used measurement technique in paediatric research for bone density and body composition assessment. This can be attributed to both the increase in the prevalence and identification of childhood diseases related to body composition and to the advances in technology around paediatric measurements. The iDXA is the latest densitometer to come from the GE Lunar range and has demonstrated improved precision and accuracy in adults compared to its predecessors; the GE Lunar Prodigy and other instruments*(**4**).* When research studies span several years or cross several research sites it may be necessary to perform measurements on different densitometers. The application of cross-validation equations across instruments can help reduce the inter-instrument error of this process on the data. There is currently no body composition phantom which can be used to compare instruments and so repeated measurements in human subjects are necessary to develop cross-validations. We have previously documented the differences between the GE Lunar iDXA and Prodigy instruments in an adult population *(**4**)* and highlighted a significant positive bias between iDXA and the 4- component (4-C) model derived FM *(**5**).* In this paper we explore the differences between iDXA and 4-C measured FM and FFM in a paediatric population.

DXA is considered a pseudo 3 component model, which separates the body into bone mineral, lean soft tissue (LST) and FM. At any site it distinguishes between 2 components: fat *vs* LST or soft *vs* bone. In the determination of body composition from body volume and body mass; the proportion of fat in weight is calculated on the assumption that the densities of fat and lean tissues are constant. Fat has relatively uniform physical properties throughout the life course (0 water content and a density of 0.9 kg/L) *(**6**).* In contrast, it has long been established that lean tissue hydration in children decreases with maturation *(**7**)* as lean tissue density increases (1.063–1.10 kg/L) *(**8**),* displaying a curvilinear response to age *(**9**).* However, a limitation of DXA estimated soft tissue is that it assumes a uniform hydration of 0.73 across both adults and children (10). Hydration of LST can be assessed through a variety of methods, varying in accuracy; deuterium dilution *(**11**).* bioelectrical impedance *(**12**)* or simply using published age and gender hydration factors such as Lohman *et al (**13**)* for paediatrics.

The aims of the present study are therefore (1) to assess the agreement of two DXA systems (GE healthcare Lunar iDXA and Prodigy) for determining total and regional (arms, legs, trunk) fat, lean, and bone mass and (2) to compare lean soft tissue hydration correction methods to iDXA LST in a healthy paediatric population.

## Methods

### Participants

One hundred and twenty four boys and girls aged between 6 and 16 years old free from disease and medications took part in the study between July 2014 and December 2016. After reading the relevant information leaflets and having the study fully explained the children then assented and parents consented to taking part in the study. The children were recruited locally through advertisements and radio and approval was granted by the Cambridge South ethics committee (REC 14/EE/0092).

Each participant arrived at the NIHR Cambridge Clinical Research Facility, Addenbrookes Biomedical Campus, Cambridge, United Kingdom, at noon on the day of their visit to undertake total body water determination using deuterium dilution, DXA, and body volume using air displacement plethysmography (ADP). The participants wore light metal-free clothing and refrained from food and drink for 30 min before and during the measurements.

### Anthropometry

Height was measured on a stadiometer and recorded to the nearest millimetre (SECA electronic stadiometer) and weight was measured on electronic scales to the nearest 100 gram (BODPOD, Cosmed Srl, Rome, Italy). Weight, Height, and BMI SDS was calculated using the LMS method (14).

### Dual Energy X-Ray Absorptiometry

The participants had 2 whole body dual energy DXA assessments for bone density and body composition (FM and LST), one scan on each GE Lunar Prodigy and iDXA (analysed in version 16, enhanced mode). Calibration block quality assurance and encapsulated spine phantom quality control scans were performed on each instrument at the start of each scanning day. The scans were performed by three trained operators (one operator for both scans per child) who performed scans according to the manufacturers positioning and scanning protocols (precision for each operator has been previously published *(**4**)*). Subsequent analysis of all scans was carried out by a single operator to ensure consistency throughout the study. A precision from BMC from iDXA (CV%) of 0.4 % was used from our previous data in adults *(**4**).*

### Body volume by ADP

Body volume (BV) was measured by ADP (BODPOD, Cosmed Srl, Rome, Italy). The participants were asked to pass urine before the procedure. Tight fitting swim wear and a swimming cap were worn to minimise air trapped in clothing and hair. Raw volume, appearing briefly on the screen during the measurement, was recorded for use in further corrections. Raw BV requires correcting for thoracic gas volume (TGV) and air next to the skin (SA artefact, SAA)

ActualBV(litres)=rawBV+0.4TGV−SAA

where TGV was predicted using child-specific equations for boys and girls (15, 16, 17) as the sum of functional residual capacity (FRC) and log tidal volume (TV).

BoysFRC(litres)=(0.02394xHeight)−1.716

$$Boys\;Log\;TV=\left(1.8643\;x\;Log_{10}\;Height\right)-1.3956$$

$$Girls\;FRC\;\left(litres\right)=\left(1.1478xHeight\right)-\left(0.0136745\;x\;Height^{2}\right)+\left(6.98227757x\;10^{-5}\;x\;Height^{3}\right)-\left(1.2725216\;x\;10^{-7}\;x\;Height^{4}\right)-33.928$$

$$Girls\;Log\;TV=\left(1.8643\;x\;Log_{10}\;Height\right)-1.3956$$

where HT is in cm. SA was calculated according to Haycock et al (1978) (18) for children:

$$SA\left(m^{2}\right)=\;0.024265\;x\;Weight^{0.5378}\;x\;Height^{0.3964}$$

where Weight is in kg and Height is in cm. SAA was then calculated as:

SAA=−kxSA

Where k is a constant from the manufacturer (Life Measurements Instruments) relating to surface area.

Precision (CV%) for BV using BODPOD was 0.3 %.

### Deuterium Dilution for total body water

A baseline saliva sample was obtained from the participant shortly after arrival. Weight was measured on electronic scales to the nearest gram (BODPOD, Cosmed Srl, Rome, Italy) for dose calculation only. The participant then consumed a dose of 70 mg per kg body mass of ^2^H_2_O (99.8 %, CK Isotopes Ltd., Ibstock, Leicestershire, United Kingdom) *(**19**).* Further 1 ml saliva samples were collected at 2, 3, 4, and 5 hours post dose. The saliva samples were frozen at -20°C until later analysis using dual inlet isotope ratio mass spectrometry (Isoprime, GV Instruments, Wythenshawe, United Kingdom).

Total body water (TBW) was calculated according to the method of the International Atomic Energy Agency *(**19**).* In brief, aliquots of 0.2 ml, drawn from the saliva samples were placed in 3.7 ml glass bottles with rubber septa (nonevacuated vials, Labco Ltd, Lampeter, United Kingdom) and equilibrated with hydrogen in the presence of a platinum catalyst. Data was drift corrected off-line and all measurements were made relative to Vienna standard mean ocean water (V-SMOW) using laboratory standards traceable to the international standard.

^2^H_2_O dilution space was determined using the following Eq. *(**20**)*:

$${}^{2}H_{2}O\;\left(kg)\right)=\left[\frac{D\times T\times \left(Ed-Et\right)}{d\times \left(Es-Ep\right)}\right]/1000$$

Where: *D* is the amount of oral dosing solution, in grams, administered to the subject; *T* is the amount of deionised tap water used to dilute the enriched isotope dose, in grams; d is the amount of enriched isotope dose in grams.

*Ed* is enrichment of the diluted dose d in T; *Et* is the enrichment of the tap water diluent; *Es* is the mean enrichment of saliva samples at 2, 3, 4, and 5 hours; *Ep* is the enrichment of the pre dose sample. TBW (kg) was then calculated by reducing ^2^H_2_O dilution space values by 4 % to account for the exchange of deuterium with non-aqueous hydrogen *(**21**).* Precision for the TBW method was determined using the 4 and 5 h samples and was 1.19 % (CV%)

### Four-component model (4-C)

FM (kg) using the 4-C model was calculated using the following equation of Fuller *et al (**5**)*:

FM=2.747×BV−0.710×TBW+1.460×BMC−2.050×Weight

Where: BV is body volume and determined using ADP; TBW is total body water and determined using deuterium dilution; BMC is whole body bone mineral content and determined using DXA and Weight is body weight determined during the ADP procedure. Precision of the 4-C model, determined using propagation of error, using precision data from the individual components (0.3 % volume, 1.19 % TBW and 0.4 % BMC), was 1.29%. We did not have precision data for weight.

### Correction of lean soft tissue (LST)

Corrected iDXA LST = iDXA LST / 0.732 * HFFM_TBW_

Corrected iDXA FFM = iDXA LST / 0.732 * HFFM_TBW_ + iDXA BMC

### Statistical analysis

Descriptive data are reported as mean ± (standard deviation) unless otherwise stated for all complete-case participants.

Paired sample *t*-tests were performed to determine the difference between instruments for all variables and hydration correction methods for FM and LST.

Bland-Altman analysis was performed to determine the association and agreement between the 2 instruments and between each instrument and 4-C derived FM and LST. Where appropriate linear regression analysis was used to derive cross-calibration equations to convert Prodigy data to iDXA data.

GraphPad Prism (Version 8.00 for Windows, GraphPad Software, San Diego California USA) was used to generate Bland-Altman analyses and IBM SPSS (IBM Corp. Released 2012. IBM SPSS Statistics for Windows, Version 25.0. Armonk, NY: IBM Corp) was used for all other statistical analyses.

Significance was assumed at *p < 0.05*.

## Results

### Cross-Calibration of DXA Data

There were significant sex differences in height, TBW, iDXA fat, lean, and bone mass and fat and FM index (FM index = 4-C FM / Height^2^). It was observed that girls were of shorter stature and had lower total body water, lean and bone masses and FFM index (FFM index = 4-C FFM/ Height^2^) than boys. In contrast, girls had a higher FM and FM index (Table 1).

**Table 1:** Characteristics of the Study Population

	Girls (*n* = 70)		Boys (*n* = 54)	
n = 124	Mean ± SD	Range	Mean ± SD	Range
Age (years)	11.4 ± 3.1	6.2 – 17.0	12.2 ± 3.0	6.5 – 16.9
Height (cm)	148.0 ± 17.7*	110.7 – 177.2	155.9 ± 19.9	119.3 – 188.2
Height SDS	0.53 ± 1.08	-1.73 – 3.56	0.69 ± 0.89	-1.51 – 2.48
Mass^a^ (kg)	42.3 ± 14.6	20.1 – 84.0	46.6 ± 16.3	19.7 – 78.8
Mass SDS	0.39 ± 1.03	-1.90 – 2.33	0.52 ± 0.99	-1.91 – 3.11
BMI (kg/m^2^^b^)	19.0 ± 3.2	13.8 – 28.9	18.8 ± 2.9	14.0 – 29.1
BMI SDS	0.28 ± 1.08	-1.99 – 2.26	0.29 ± 1.20	-3.01 – 3.12
TBW (kg)	23.2 ± 7.3*	11.7 – 41.3	28.1 ± 10.2	14.0 – 48.6
4-C Fat (kg)	10.8 ± 6.0	2.9 – 28.8	8.6 ± 4.7	2.3 – 25.7
4-C FFM (kg)	31.5 ± 10.3	16.1 – 55.2	38.1 ± 14.5	18.1 – 70.1
Raw body volume a (L)	38.9 ± 13.9	18.2 – 80.0	42.2 ± 14.7	19.4 – 74.8
Fat mass^b^ (kg)	12.6 ± 6.0*	4.3 – 32.7	10.7 ± 4.6	3.8 – 26.9
Lean mass^b^ (kg)	28.7 ± 9.3*	13.1 – 49.3	34.8 ± 13.1	17.1 – 62.3
Bone^b^ mass (kg)	1.60 ± 0.57*	0.75 – 2.95	1.89 ± 0.77	0.88 – 3.44
FMI c (kg/m^2^^b^)	4.8 ± 2.2*	1.9 – 10.0	3.6 ± 1.9	0.9 – 9.5
FFMI c (kg/m^2^^b^)	14.0 ± 1.7*	11.1 – 18.9	15.0 ± 2.2	11.9 – 20.1
Hydration (%FFM)	74.7 ± 0.03	65.0 – 82.0	74.1 ± 0.02	69.0 – 79.0

BMI, body mass index; FFM, fat free mass; FM, fat mass; FMI, fat mass index; FFMI, fat free mass index; SDS, standard deviation score; TBW, total body water; 4-C, four-component.

^a^ BODPOD.

^b^ iDXA.

^c^ 4 component model.

⁎ significantly different from boys *p* < 0.05.

**Fig. 1:**
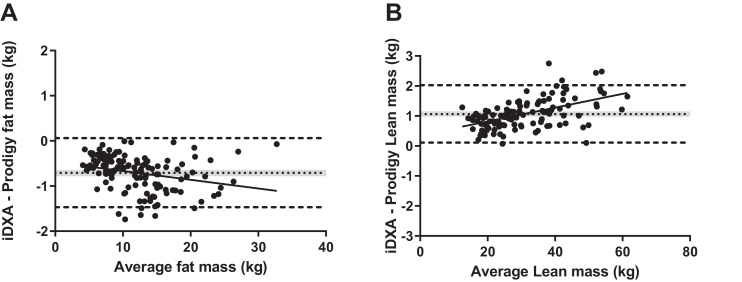
Bland-Altman analysis of the agreement between iDXA and Prodigy measured fat mass (A) and lean soft tissue mass (B) in children.

Table 2 presents the biases and limits of agreement between iDXA and Prodigy whole-body and regional bone and body composition measurements. Although all variables were significantly and linearly correlated, significant differences exist between all variables, with the exception of total-body, leg, and trunk bone mineral content (BMC). The Bland-Altman plots displayed (Fig. 1), for the variables with the largest absolute differences, demonstrate a bias between the scanners, with iDXA measuring a 0.71 kg (6 %) lower total body FM and a 1.07 kg (3.5 %) higher total body LST than Prodigy.

**Table 2:** Bland-Altman Analysis of the Agreement Between iDXA and Prodigy Regions of Body Composition in the 124 Study Participants

(kg)	iDXA	Prodigy	Bias	Sig	LOA
Total fat mass	11.8	12.5	-0.71	<0.05	-1.48, 0.06
Arm fat mass	1.4	1.6	-0.12	<0.05	-0.33, 0.08
Leg fat mass	5.1	5.4	-0.34	<0.05	-0.80, 0.12
Trunk fat mass	4.5	4.8	-0.25	<0.05	-0.74, 0.23
Total LST	31.4	30.3	1.07	<0.05	0.11, 2.03
Arm LST	3.1	2.9	0.16	<0.05	-0.12, 0.44
Leg LST	11.0	10.4	0.63	<0.05	0.04, 1.22
Trunk LST	14.5	14.3	0.23	<0.05	-0.61, 1.07
Arm bone mass	0.204	0.202	0.002	<0.05	-0.01, 0.02
Leg bone mass	0.643	0.644	-0.001	ns	-0.03, 0.03
Trunk bone mass	0.494	0.494	-0.000	ns	-0.04, 0.04
Total BMC	1.729	1.723	-0.001	ns	-0.06, 0.06

LST, lean soft tissue; BMC, bone mineral content; LOA, limits of agreement.

Cross calibration equations were developed for all variables (Table 3) with the exception of total-body, leg and trunk BMC.

**Table 3:** Cross Calibration Equations Between Prodigy and iDXA Measurements for the 124 Study Participants

	Slope	CI	Intercept	CI	r^2^
Total fat mass	0.979	0.967, 0.991	-0.442	-0.607, -0.277	0.995
Arm fat mass	0.951	0.922, 0.979	-0.047	-0.095, 0.001	0.972
Leg fat mass	0.970	0.953, 0.988	-0.181	-0.283, -0.079	0.990
Trunk Fat mass	0.995	0.979, 1.012	-0.232	-0.323, -0.140	0.991
Total LST	1.023	1.017, 1.030	0.363	0.146, 0.579	0.999
Arm LST	1.018	1.000, 1.037	0.111	0.052, 0.170	0.990
Leg LST	1.029	1.018, 1.041	0.323	0.194, 0.451	0.996
Trunk LST	1.015	1.001, 1.029	0.014	-0.201, 0.229	0.994
Arm bone mass	1.002	0.989, 1.016	0.001	-0.002, 0.004	0.994

LST, lean soft tissue; CI, confidence intervals; r, correlation coefficient.

### Four-component (4-C) model comparison to iDXA

There were significant differences between iDXA measured and 4-C derived FM and FFM in children (Table 4), with iDXA significantly over estimating FM by 2 kg and under estimating FFM by 1.3 kg. The bias in FM was unrelated to total FM, however, in the case of FFM the bias was positively related to total FFM. There were no sex related differences.

**Table 4:** Differences Between the Four Component Model Calculated and iDXA Measured Fat and Fat-Free Mass

	4-C	iDXA	Bias	CI	Sig	R
Total FM (kg)	9.8	11.8	2.0	2.2 to 1.7	P < 0.05	0.960
Girls	10.8	12.6	1.8	2.2 to 1.4	P < 0.05	0.959
Boys	8.6	10.7	2.1	2.5 to 1.8	P < 0.05	0.958
Total FFM (kg)	34.4	33.1	-1.3	-1.1 to -1.5	P < 0.05	0.996
Girls	31.5	30.3	-1.2	-1.0 to -1.5	P < 0.05	0.994
Boys	38.1	36.7	-1.4	-1.0 to -1.7	P < 0.05	0.997

FM, fat mass; FFM, fat-free mass; 4-C, four component model; CI, confidence interval; R, correlation coefficient

In order to determine if hydration was an underlying factor between the differences, we calculated individual fat free mass hydration factors (HFFM) based on TBW measurements and 4-C derived FFM and corrected the iDXA estimations. We compared this to iDXA corrections made using the published paediatric hydration coefficients of Lohman.

**Fig. 2:**
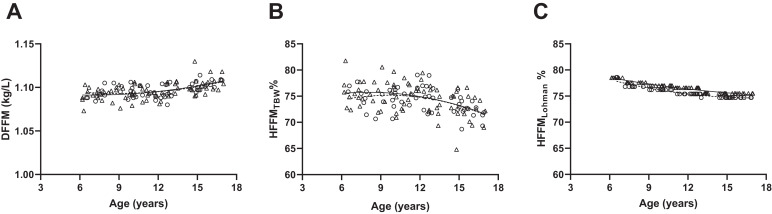
Density (A) and hydration (B) of fat free mass calculated by TBW or Lohman (C) coefficients in 124 healthy boys (open circles and dashed line) and girls (open triangles and solid line). DFFM, density of fat free mass; HFFM, hydration of fat free mas; TBW, total body water.

**Fig. 3:**
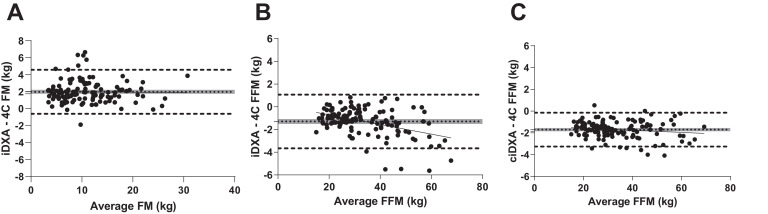
Bland-Altman analysis of the agreement between 4C calculated and iDXA measured and iDXA corrected (ciDXA) fat (A) and fat free mass (B & C) in children. Mean and limits of agreement. FM, fat mass; FFM, fat free mass; 4-C, four-component model.

There were significant differences between HFFM_TBW_ compared to HFFM_Lohman_. Figure. 2 illustrates the different relationship HFFM has with age and sex between the 2 coefficients. It is apparent that when calculating HFFM_TBW_ there is a large variation within an age group which is not accounted for within the age specific Lohman coefficients. It was observed that the HFFM_TBW_ were consistently lower for each age group compared to HFFM_Lohman_ (Table 5). When iDXA FFM was corrected for hydration using HFFM_TBW_ the limits of agreement were reduced and the bias no-longer displayed a positive relationship with total FFM (Fig. 3). When iDXA LST was corrected using the two different HFFM methods, significant differences between mean uncorrected LST and corrected LST were observed (Table 6).

**Table 5:** Comparison Between Lohman Hydration Factors and TBW Hydration Factors

	Girls		Boys	
Age (years)	Lohman (%)	Watson (%)	Lohman (%)	Watson (%)
5 - 6.99	78.0	75.7 ± 3.1	77.0	74.8 ± 2.8
7 - 8.99	77.6	75.4± 2.0*	76.8	74.2 ± 2.5*
9 - 10.99	77.0	75.2 ± 3.1*	76.2	74.7 ± 1.8*
11 – 12.99	76.6	76.0 ± 2.0	75.4	75.1 ± 2.4
13 – 14.99	75.5	73.1 ± 3.3*	74.7	74.1 ± 1.8
15 – 16.99	75.0	72.5 ± 2.6*	74.2	72.2 ± 1.7*

⁎ Significantly different to Lohman. Age; 5–6.99 years, *n* = 7 (girls), 2 (boys); 7–8.99, *n* = 11 (girls), 7 (boys); 9–10.99, *n* = 15 (girls), 11 (boys); 11–12.99, *n* = 14 (girls), 14 (boys); 13–14.99, *n* = 13 (girls), 7 (boys); 15–16.99*, n* = 10 (girls), 13 (boys).

**Table 6:** Differences in Lean Soft Tissue When Corrected for Hydration Factors

	Uncorrected iDXA^1^	Corrected using TBW HFFM^2^	Corrected using Lohman HFFM^3^	Difference		
				1-2	1-3	2-3
Girls						
LST (kg)	28.7	28.2	27.5	0.5*	1.2*	0.7*
HFFM (%)	73.0	74.6	76.7			
Boys						
LST (kg)	34.8	34.5	33.8	0.3*	1.0*	0.7*
HFFM (%)	73.0	74.1	75.7			

HFFM, hydration fat free mass; LST, lean soft tissue; TBW, total body water.

^1^ Uncorrected iDXA.

^2^ Corrected using TBW HFFM.

^3^ Corrected using Lohman HFFM.

⁎ *p* < 0.05.

## Discussion

The primary aim of this study was to expand on our previous research in adults and investigate the differences between the GE Lunar iDXA and GE Lunar Prodigy DXA scanners in a healthy paediatric cohort. Although we observed strong linear relationships between the 2 systems, significant differences existed in whole body bone masses and body composition and therefore cross-calibration equations were derived.

The largest differences observed were in total body FM and LST, with iDXA under estimating total body fat by 6 % (0.71 kg) and over-estimating LST by 3.5 % (1.07 kg) compared to Prodigy, consistent with our adult findings (4). A recent study by Crabtree *et al (**22**)* performed cross-calibration of 70 children aged 5–20 years and the findings in their supplementary material are similar to the cross-calibration equations for soft tissue (using encore software version 15 and 16) in the current study (total lean mass = slope 1.001, intercept 0.521 compared to 1.023 and 0.362 and total FM = slope 0.992, intercept -0.159 compared to 0.979 and -0.442). However, unlike Crabtree *et al*, we did not derive cross-calibration equations for total BMC between the 2 systems as we did not observe significant differences between the scanners for these variables. This may have been due to the later version of software (encore 16) being used in our analysis on both scanners.

The technical differences between the two models of GE Lunar has been previously discussed *(**4**,* *23**).* In brief, the main differences between the scanners are a change in the detector and X-ray filter, within the iDXA which provide an improved resolution and image quality, resulting in an enhancement of the bone edge detection technology. Therefore these technical advances in bone edge detection have encouraged the development of the soft-tissue algorithms to predict lean and FM. However, further fine tuning of the algorithms within software versions are creating differences in lean and FM prediction within the GE Lunar iDXA model.

The secondary aim was to explore the agreement between iDXA soft tissue estimates and those of the 4-C model. The overestimation of FM by DXA in children compared to the 4-C model is historical, with several studies reporting the overestimation of FM measured by Lunar Prodigy DXA compared to the 4-C model across children and adults *(**4**,* *24**,* *25**).* In agreement with these previous studies, we observed that GE Lunar iDXA significantly over estimated FM compared to the 4-C model. Further, the difference observed was larger in children compared to our previous study in adults (2.0 kg vs 0.9 kg in children and adults respectively). In contrast to the over estimation in FM, we observed a significant under estimation in iDXA measured FFM compared to the 4-C model. With a clear bias observed between body size and accuracy of FM measurements by DXA, this may suggest that tissue depth has an impact on bone edge detection and therefore the point at which soft-tissue is determined.

Another possible reason for the differences observed between iDXA and the 4-C model, which has previously been suggested, *(**4**)* is hydration. The 4-C model takes total body water into account when predicting FM, however this is not the case with iDXA. A novel aspect of this study was the correction of DXA estimated LST using stable isotope derived hydration factors (HFFM_TBW_). We used the hydration factors to correct iDXA estimates of LST instead of accepting the uniform hydration factor of 0.73 embedded within the encore software and used across adults and children. When individual hydration factors were calculated for children there was a diverse and varied relationship between HFFM and age. The relationship between age and HFFM in our study was found to be curvilinear, with the decline in HFFM occurring around 12 years of age and at the point where the gender difference disappears. The variation in HFFM is in agreement with a recent study by Gutiérrez-Marin *et al*. who went on to determine that approximately 30 % of the variability in HFFM can be attributed to age and BMI *(**26**).* This supports the argument that a linear assumption of 0.73 across children and adults is inappropriate. More commonly used than deriving one's own hydration coefficients, is the use of the age and sex specific Lohman hydration coefficients *(**13**)* for children. The work of Lohman supports the curvilinear relationship between age and HFFM, however the categorization of ages disguises the large variation observed in HFFM within an age group and between the sexes, presented by a much shallower curve. In agreement with our study, Wells *et al (**27**)* have shown hydration values ranging from 76% to 73% in children from age 5 to 20 years, where hydration coefficients in the girls also differed significantly to Lohman. Although assessing an individual HFFM will always be more accurate, this is not always practical and therefore the use of previously reported values offers an alternative and allows for some variation in HFFM to be accounted for and applied to DXA LST.

Body composition assessments lose accuracy in conditions such as disease, puberty, and obesity due to the variability of hydration in lean tissue and the tendency to overestimate FM in DXA. Unfortunately puberty was not accounted for in the current study, however it would be a valuable addition to future research when accounting for differences in hydration with growth and maturation. When studying conditions outside of the norm, high levels of accuracy are required. Conditions such as lipodystrophy, in which patients present with elevated LST and markedly reduced FM, may shift the extra- to intra-cellular fluid ratio compared to controls *(**28**)* and in high levels of adiposity where hydration can increase by 2% could contribute towards the inaccurate categorisation of obesity *(**29**).* DXA is, therefore, limited due to the assumption of constant hydration values for LST and multicomponent models are preferred especially in disease states where tissue hydration is likely to be altered.

In conclusion, we have shown that cross-calibration equations are necessary to correct FM and LST Prodigy data to fall in line with iDXA data. Our study also provides evidence of the limitations of DXA alone when interpreting body composition in children, due to the overestimation of FM and under-estimation of FFM, possibly due to variation in LST hydration across and within age groups or other factors. Care should be taken to fully understand the measurements and algorithms used within paediatrics, especially when analysing participants that do not fall within a typical range.
